# Insights into the Pathophysiology of Infertility in Females with Classical Galactosaemia

**DOI:** 10.3390/ijms20205236

**Published:** 2019-10-22

**Authors:** Zaza Abidin, Eileen P. Treacy

**Affiliations:** 1 National Centre for Inherited Metabolic Disorders, Adult Services, Mater Misericordiae University Hospital, Dublin, Ireland; 2 Department of Paediatrics, Trinity College Dublin, Dublin, Ireland; 3 School of Medicine and Medical Sciences, University College Dublin, Dublin, Ireland

**Keywords:** classical galactosaemia, primary ovarian insufficiency, pathophysiology, fertility preservation

## Abstract

Classical galactosaemia (CG) (OMIM 230400) is a rare inborn error of galactose metabolism caused by the deficiency of the enzyme galactose-1-phosphate uridylyltransferase (GALT, EC 2.7.7.12). Primary ovarian insufficiency (POI) is the most common long-term complication experienced by females with CG, presenting with hypergonadotrophic hypoestrogenic infertility affecting at least 80% of females despite new-born screening and lifelong galactose dietary restriction. In this review, we describe the hypothesized pathophysiology of POI from CG, implications of timing of the ovarian dysfunction, and the new horizons and future prospects for treatments and fertility preservation.

## 1. Introduction

Classical galactosaemia (CG) (OMIM 230400) is a rare inborn error of galactose metabolism caused by deficiency of the enzyme galactose-1-phosphate uridylyltransferase (GALT, EC 2.7.7.12), the second enzyme in the Leloir pathway, the pathway for galactose metabolism [1]. Classical galactosaemia is also known as Type 1. Type 2 galactosaemia is caused by mutation of the *GALK1* gene, characterized by deficiency of galactose kinase 1 enzyme. Type 3 is caused by mutations involving the *GALE* gene, characterized by deficiency of the enzyme UDP-galactose-4-epimerase. Recently, Wada et al. [2] described novel finding pathogenic variants of the *GALM* gene which encodes galactose mutarotase, the enzyme which catalyzes the epimerization between β and α-D-galactose in the first step of the Leloir pathway in patients with ‘unexplained congenital galactosaemia. This has suggested a new Type IV classification for galactosaemia [2].

The prevalence of classical galactosaemia (CG), Type 1, ranges from 1:16,000 to 1:60,000 in Europe and USA [3,4]. The incidence in the Irish population is 1:16,476 and is 1:430 in the Irish Traveller community [4]. Although classical galactosaemia has been described since 1908 and the gene identified in 1992, it is still considered a partially treated rare disease. Restriction of galactose is life-saving in the neonate and improves the neonatal intoxication manifestations of feeding difficulties, failure to thrive, sepsis, hepatocellular damage, renal tubulopathy, and cataracts. Affected individuals develop long term complications affecting the central nervous system, bone density/metabolism, and primary ovarian insufficiency and subfertility in females despite dietary galactose restriction [4,5,6,7,8,9]. Primary ovarian insufficiency in CG, first described in 1978 [10] with ovarian follicular depletion is reported in at least 80% of females [9]. This is a particularly devastating complication for affected females. Moreover, the timing of the ovarian insult and its pathophysiology are poorly understood, which limits interventions. This article aims to clarify recent insights into the pathophysiology of this presentation, and the consideration of new therapeutic interventions.

## 2. Methods

Literature on the effects of CG on the female reproductive system was reviewed by an extensive Pubmed search (publications from January 1975 to September 2019) using the keywords: galactosaemia; ovarian function/dysfunction; primary ovarian insufficiency/failure; follicle stimulating hormone (FSH); oxidative stress; fertility preservation. In addition, articles cited in the search articles and literature known to the authors were also included in this review. A review of the Cochrane database was also performed with the condition ‘Galactosaemia.’ This did not indicate any review relating to infertility in females with CG.

## 3. Background

### 3.1. Galactosaemia and Galactose Metabolism

**Figure 1:**
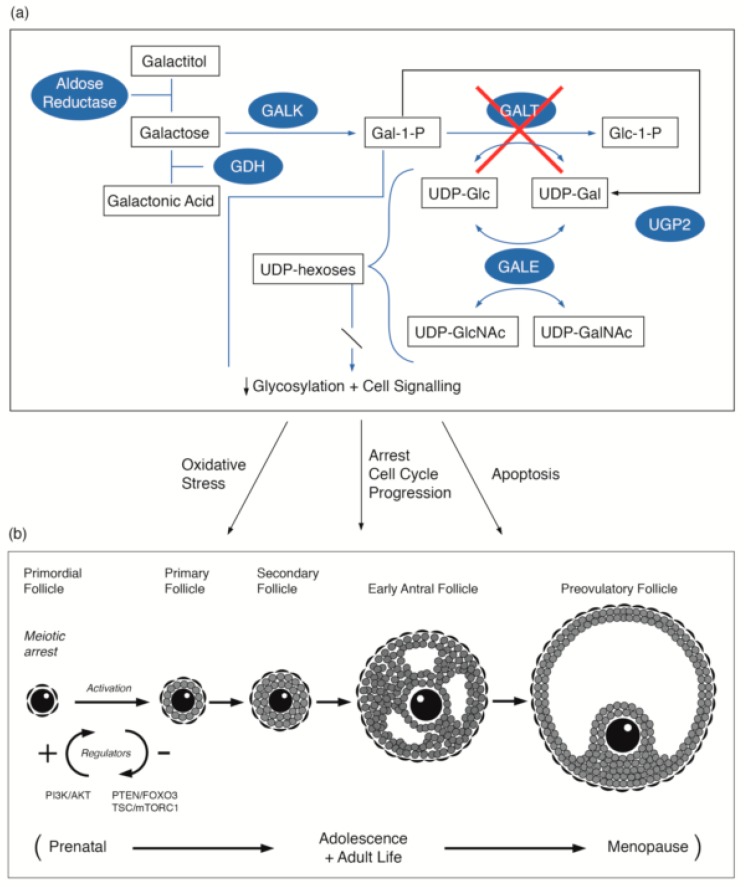
(**a**) Illustration of the pathways of galactose metabolism, with inhibition of the key enzyme: galactose-1-phosphate uridylytransferase. The key enzymes involved are shaded. (**b**) Illustration of the steps in oogenesis and folliculogenesis over the prenatal to menopause time period that may be influenced by GALT deficiency [16,17,18,19,20] with the proposed site of the PI3K/AKT regulation as cited by Sanchez and Smitz [20]. The abbreviations used are listed in the abbreviations list.

Galactose is converted to glucose-1-phosphate and metabolized to release energy, or alternatively, galactose may be metabolized to UDP-galactose (UDP-GAL) and its derivatives. UDP-GAL is an essential cofactor for the galactose transferases that are involved in the incorporation of galactose into glycoproteins and glycolipids [11]. Galactosaemia is an inborn error of metabolism resulting in impaired activity of one of the four enzymes involved in the main galactose metabolism pathway (known as the Leloir pathway): galactokinase (GALK), galactose-1-phosphate uridylyltransferase (GALT), UDP galactose 4-epimerase (GALE) and the recently described galactose mutarotase (GALM) deficiency, (Figure 1a). Deficiency in GALT causes classical galactosaemia Type 1 (CG). Although the liver is the major organ for galactose metabolism, the enzymes of the Leloir pathway have been found in many cell types and tissues, including the gonads, intestinal mucosa, kidneys, skeletal muscles, fibroblasts, leukocytes, and red cells. Galactose-1-phosphate uridylyltransferase and galactokinase are present in fetal red cells, the liver, the lung, the spleen, and cardiac muscle from at least 10 weeks’ gestation, and their activities are higher in the second and third trimesters than at any time postnatally [12]. This suggests the possibility that damage may occur in utero in GALT deficiency. Studies in rat tissue showed the liver to have the highest GALT mRNA and GALT activity. Kidneys, ovaries, and the heart have similar but lower mRNA and GALT activities, and skeletal muscle and testes have the least [13]. The long-term complications of CG are known to be present in organs with high physiological GALT-expression. Endogenous galactose production occurs in utero and throughout life. It is age-related, with higher levels in children than in adults. The endogenous production of circulating free galactose in adults ranges from 0.53 to 1.05 mg/kg/h [14,15].

Classical galactosaemia represents the most severe form of this disorder as a result of profound impairment in *GALT* resulting in accumulation of galactose, galactose-1-phosphate, galactitol, and galactonate in body tissues and fluid. Over 300 different mutations are described in the *GALT* gene [21]. The commonest genotype in European populations is the homozygous *GALT* Q188R genotype which involves the enzyme’s active catalytic site and is associated with a severe phenotype [22]. The S135L variant, predominantly found in the African and African-American populations, results in significant GALT residual activity and a generally milder phenotype [23].

### 3.2. Primary Ovarian Insufficiency in Female Galactosaemia Patients

Primary ovarian insufficiency is considered to be present when a woman who is less than 40 years old has had amenorrhea for 4 months or more, with two serum FSH levels (obtained at least 1 month apart) in the menopausal range [24]. This may present as delayed puberty, oligomenorrhoea/amenorrhoea or irregular menstrual cycles, progressing to infertility and complications of hypoestrogenism.

The association between galactosaemia and primary ovarian insufficiency (POI) was first reported in 1979 [25].

The Galactosaemia Consortium (GalNet) Registry outcome study [9] has recently demonstrated that 80% of females (of *n* = 164) have primary ovarian insufficiency which is consistent with other studies [4,5,6,7,8,26,27].

The onset and timing of damage causing primary ovarian insufficiency (POI) in CG patients remain unknown. It is unclear at which stage of development this occurs, and whether this is ongoing due to galactose exposure or a single event leading to irreversible life-long damage. In depth understanding of the pathophysiology and the mechanism of this process and the implications will lead to the possibility of reversing or even stopping this process. Risk factors for developing ovarian insufficiency in CG include homozygosity for the *GALT* Q188R mutation, severe whole-body galactose oxidation impairment, and mean Gal-1-P levels in erythrocytes [28]. There were no correlations found between the age of initiation of dietary galactose restriction, compliance, or high erythrocyte Gal-1-P levels with POI from CG.

Females with CG generally have higher concentrations of FSH and reduced concentrations of anti-mullerian hormone (AMH) [29,30]. In total, 90% of patients showed signs of hypergonadotropic hypogonadism over the 4-year study period in the study of Kaufman et al. 1986 [31].

It is proposed that the ovarian damage occurs prenatally, suggested by reduced oocyte numbers in rats subjected to prenatal exposure to high levels of galactose [32]. Streak ovaries have been reported on laparoscopy in young females [25,33,34,35,36]. Postnatal damage is supported by the report of normal ovarian tissue in a girl who died from *Escherichia coli* sepsis at 5 days of age [37], and by the finding that in some patients, gonadal function may initially be normal but can become abnormal with time [31]. Ovarian histology in galactosaemic women with ovarian insufficiency has been variable. The numbers of oocytes have reportedly been absent or low, with fibrous and streaky stroma. In a study of 53 girls with galactosaemia, 77% experienced POI at a mean age of 13 years [28]. A case report of a 17-year-old girl with CG indicated that at the age of seven, her ovaries were normal upon abdominal exploration, but at age 17 the ovaries were described as streaked in appearance [25]. Several other case reports described small, atrophic, streak-like, and sometimes undetectable ovaries in patients between ages of 13 and 30 years [38]. Mamsen et al. studied the pathology of six CG prepubertal girls below age 12 (mean age 3.8 ± 1.7 years; range 0.4–11.7 years)). All five girls less than age five had follicle counts within the normal control limits. In contrast, there were no follicles detected in the sample taken from the 11.7-year-old girl [39].

Although the prevalence of spontaneous conception in POI was previously considered to be low [1], Van Erven et al., 2017 reported that nine out of 21 patients with CG galactosaemia achieved pregnancy (42.9%) [40]. This pregnancy rate was higher than anticipated based on reported cases of POI from any cause (5–10%).

Hypergonadotropic hypogonadism is usually diagnosed in the second decade of life in women with CG. The serum FSH levels may be noted to be elevated, however, as early as 4 months and from early childhood to the onset of puberty. LH levels may be variable; however, oestradiol is usually low [1,24].

The exact mechanism underlying POI from CG is still unknown. There have been a number of possible pathogenic mechanisms proposed. One is the direct toxic effects of galactose and its metabolites to the ovary, and ovarian development. Another hypothesis is atypical function of FSH and/or its receptor secondary due to glycosylation abnormalities. Additionally, cell signalling abnormalities, epigenetic mechanisms, and premature apoptosis have been proposed [41,42,43].

The relatively high physiological GALK, GALT, and GALE activities in the ovaries cause the accumulation of galactose and its metabolites. Direct toxic damage to the ovary through either galactose or its metabolites may occur. Galactitol if not metabolized causes accumulation in ovarian cells, leading to swelling and cell dysfunction, with oxidative stress and premature apoptosis [43].

The activity of UDP-glucose (UDP-Glc) pyrophosphorylase highly expressed in ovarian tissue is critical for nucleotide sugar production and maintenance of germ cell function, follicular maturation and steroidogenesis. UDP galactose (UDP-Gal) is required for the synthesis of glycoproteins and galactolipids that serve many functions; for example, in the ovarian membrane, the support of germ cells, follicular maturation, and steroidogenesis. Therefore, UDP-Gal deficiency might be a pathogenic mechanism [1]. This mechanism has not been studied, however, in regard to ovarian function. Recently, Rubio-Gozalbo et al. 2019, identified abnormal uridine sugars in the zebrafish galactosaemia model. The result from their study showed that CG zebrafish have a decreased UDP-Glc:UDP-Gal ratio in the ovary with a lower UDP-GlcNAc:UDP-GalNAc ratio compared to the wild-type zebrafish. CG fish also showed increased CMP-Neu5Ac levels, suggesting impaired sialyation and increased GDP-fucose levels in early developmental stages and in adulthood, particularly in the ovary, pointing to fucosylation impairments [44].

It was also proposed that auto-ovarian antibodies could cause ovarian insufficiency in CG. This mechanism has not been confirmed, however, in CG.

Earlier studies have reported normal FSH bioactivity], but this does not exclude abnormalities of the FSH-FSH receptor interaction secondary to hypoglycosylation [45,46].

Our group has described significant systemic n*-*glycosylation deficiencies in CG children and adults in circulating serum glycoproteins and IgG. This is best described as a mixed CDG-I and II defect, and n-glycan assembly and processing defect [47,48,49]. Fundamental findings in the measured glycans are increased fucosylation and branching defects which could potentially affect cell adhesion and cell signalling. Patients with primary congenital disorders of glycosylation (CDG), due to mutations in genes encoding for products involved in glycan assembly also present with learning impairment, speech defects, and ovarian failure with hypergonadotrophic hypogonadism, suggesting a similar pathophysiological aetiology. A similar clinical feature is also seen in female patients with an inactivating mutation of the FSH-receptor showing hypergonadotropic hypogonadism with delayed or no spontaneous puberty [50].

In addition to abnormalities of circulating glycoproteins our group Coman et al., 2010, studying T cell gene microarray expression, identified a major systemic signalling pathway dysfunction in galactosaemia patients involving aberrations of the MAPK signalling pathway, the regulation of the actin cytoskeleton, the calcium signalling pathway, the cell adhesion molecules in addition to the haematopoietic cell lineage, and cytokine-cytokine receptor interactions. This was proposed to be as a consequence of systemic abnormal galactosylation of numerous cellular glycoproteins with possible abnormalities of protein glycan site occupancy, folding, and survival [16]. A further follow up study of a larger patient sample size illustrated systemic dysregulation of numerous gene pathways, including glycosylation, inflammatory, and inositol signalling pathways [17].

In a further study from our group, we studied specific glycan synthesis, the leptin system, and inflammatory gene expression in white blood cells as the potential biomarkers of infertility in 54 adults with CG on a galactose-restricted diet in a multi-site Irish and Dutch study. Systemic dysregulation of genes, including *LEP, LEPR, ANXA1,* and *ICAM1* was evident for CG patients, and combined with abnormalities of IgG glycosylation, hormonal and leptin analyses illustrated the presence of systemic glycosylation, inflammatory, and cell signalling abnormalities [19].

A number of galactose toxicity studies in animal models are informative. The investigation of pregnant rats fed a 50% galactose diet showed a striking reduction in oocyte number in the offspring [32]. Additionally, adult female mice fed a 50% galactose diet demonstrated a decrease in ovulatory response [51].

Bandyopadhyay et al., 2007 conducted a study of feeding pregnant rats with 35% galactose supplements from day 3 of conception continuing through weaning of the litters. The induced galactose toxicity in the offspring delayed the onset of puberty in the female rat offspring and the offspring rats subsequently developed hypergonadotrophic hypoestrogenism [52].

A *GALT* knockout zebrafish model has been successfully developed to mimic the human phenotype at both the biochemical and clinical levels of CG [53]. The knockout zebrafish accumulated elevated concentrations of Gal-1-P, which was exacerbated upon exposure to exogenous galactose. Knockout pairs, wildtype females/knockout males, and knockout females/wildtype males exhibited a significantly lower egg quantity per mating as compared to wildtype pairs. The observed gonadal impairments in unexposed *GALT* knockout zebra fish are in line with the human phenotype of ovarian sequelae. Impaired male infertility in fish is in line with previous reports of possible sub-clinical male infertility [54].

These clinical findings in the zebra fish model are in line with observations in a GALT-deficient mice model, whereas the females showed smaller litter size and longer time to achieve pregnancy [55]. Despite the normal ovaries on autopsy in two neonates [37,56], observations in animal models suggest a prenatal origin of the damage [32,57].

In the GALT deficient mouse model that demonstrated a subfertility phenotype in adult females, Balakrishnan et al. identified that GALT is a positive regulator of the PI3K/Akt signalling pathway [18]. The protein expression of BiP and PTEN (negative regulator) of the PI3K/Akt signalling pathway was higher in GALT deficiency. The up-regulation of PTEN was also demonstrated in the human GC study of Coss et al. (2014), as described earlier [17]. Salubrinal, a chemical compound that alleviates ER stress when administered to mutant mouse fibroblast cell lines normalised the down regulated PI3/Akt signalling pathway and the premature loss of primordial follicles seen in young mutant mice [18].

Interestingly, to date, there is no published data concerning the long-term outcome of children from mothers with classical galactosaemia. There is one case report of a healthy child born to a female CG patient who had documented premature ovarian insufficiency (POI), who conceived following FSH therapy [58].

### 3.3. Link to Primordial Germ Cell Signalling

Oogenesis, oocyte and follicular growth, and the development and oocyte maturation are complex processes regulated by intra and extra ovarian factors and signalling mechanisms, which, due to the systemic cell signalling abnormalities in galactosaemia, are proposed to be aberrant in galactosaemia. Oocytes originate from primordial germ cells in the extra embryonic mesoderm. Ovarian factors produced by theca/stromal cells, somatic granulosa cells, participate and regulate oocyte and follicle development at each of the developmental stages [20].

The first production of primordial follicles contributes to the onset of female puberty and fertility. The continuing adult production of primordial follicles provides fertility to usual female menopause. The majority of ovarian primordial follicles are maintained quiescently as a reserve for the reproductive life span. In recent years the molecular and signalling mechanisms that regulate the activation of primordial follicles have been described. Pathways, such as the phosphatidylinositol 3 kinase (PI3K) pathway with its regulator repressor protein PTEN, and the linked MTORC1 pathway and FOXO3 protein, have central roles [59,60]. FOXO3 is considered to have a repressor function. Kim et al., has confirmed the pivotal role of PTEN–PI3K signalling in the activation of primordial follicles [60]. We propose that dysregulation of the PTEN–PI3K signalling pathway in galactosaemia patients was identified in our studies and in the Gal deficient mouse model may be implicated in the primordial follicular dysgenesis observed in galactosaemia (Figure 1b) [17,18].

Recent studies have reported successful in vitro activation of mammalian primordial follicles with synthetic PTEN inhibitors [60,61]. The use of existing primordial follicles as a source for obtaining fertilizable oocytes offers a new, exciting addition for the treatment of females diagnosed with POI. Successful in vitro activation of mouse and human primordial follicles with the synergistic use of an MTORI activator and a PTEN inhibitor has now been reported [62,63].

## 4. Approaches to Treatment

### Fertility Preservation

The Galactosemia Network (GalNet) makes recommendations for girls and women with CG, for annual monitoring for menstrual abnormalities, secondary amenorrhoea, and symptoms of POI, including FSH levels. Women with hypergonadotropic hypogonadism or POI should have counselling and support on their reproductive options and management of their irregular or absent menses. A referral to a reproductive endocrinologist can be offered to women who desire pregnancy but are unable to conceive naturally or who would like to know other available fertility treatment [64].

One of the key challenges to prevention of POI from CG remains the uncertainty of the timing of the irreversible changes, which may be within the first 10 years of life with a likely prenatal effect. The current approach to treatment for galactosaemic women with POI mainly involves hormonal replacement with oestrogen supplementation and added progesterone to reduce the risk of endometrial hyperplasia or cancer.

Despite the high prevalence of POI and infertility in women with galactosaemia, spontaneous unassisted pregnancies are possible, even repeatedly. They have been reported and are not as rare as previously assumed as demonstrated by the recent study referred to earlier [40]. This should be emphasized to patients and they should not be discouraged from trying conceiving naturally. The chances of conceiving are higher in patients who reached puberty spontaneously and in younger patients, as in any woman, fertility declines with age.

A conservative approach to achieve pregnancy in POI is through ovarian stimulation. If this fails or is not possible, the next available treatment option is oocyte donation. Female CG patients have been reported to have successful pregnancy after this procedure [40]. Even though this procedure has been proven to be effective with higher pregnancy rates; the cost, availability of donor eggs, and need for psychological counselling are limiting factors. Embryo freezing after in vitro fertilization (IVF) is another available option with good outcomes. However, a major limiting factor for this procedure in this cohort of patients is that a large number of eggs would need to be retrieved from controlled ovarian hyperstimulation which may not be effective in CG females with diminished ovarian reserve.

Ovarian tissue cryopreservation and transplantation (OTCP-TP) has dramatically evolved over recent years from experimental breakthrough to a well-accepted treatment. Unlike the conservative treatment approach to POI. This procedure does not require ovarian stimulation. The techniques involve ovarian tissue extraction, freezing/thawing, and in vitro maturation or transplantation back into the same patient. This procedure is remarkably successful, with studies reporting return of ovarian function in up to 95% of graft recipients and pregnancy rates of between 30–50%. The most significant limitation of OTCP-TP is the massive loss of follicles that occurs following transplantation, which is primarily attributed to ischaemic damage and follicle activation [65].

Cutting-edge technologies for in vitro follicle growth, and tissue engineering principles to bioengineer artificial ovaries are currently being explored [66,67].

Currently, there are no defined recommendations for fertility preservation for patients with CG [68]. As the onset of ovarian reserve depletion is still unclear, this complicates the timing to offer fertility preservation. Most of the girls who would benefit from fertility preservation are too young to make the decision and to consent. Counselling female patients with CG regarding their fertility is complex with the evidence of spontaneous pregnancies and it is difficult to predict the ideal candidates for fertility preservation. The publication of van Erven et al. 2017 proposed a number of recommendations regarding fertility preservation in CG females [68]. These included encouraging patients to recognise that spontaneous pregnancies can occur in women with CG with POI, recommendations regarding ovarian cryopreservation at an early pre-pubertal age, aspects of fertility preservation, and other options.

## 5. New Horizons and Future Prospects for Fertility Treatments in Classical Galactosaemia

A major breakthrough in infertility is the activation of dormant ovarian follicles using phosphatidylinositol-3-kinase activators and the suppression of the Hippo signalling pathway—with successful pregnancies reported using this method [69,70]. This may be applicable to galactosaemia given that this pathway is involved. However, an overall approach to timely stabilisation of the function of the mutant GALT enzyme during the critical early childhood period (or indeed prenatal period) may be required to stabilise not only the galactose and its products’ intoxication effects, but also the dysfunctional systemic glycosylation defects, and subsequent systemic signalling abnormalities.

Therapeutic strategies for intervention (new treatments in development for CG), such as chaperones, antioxidants, substrate inhibitors, aldose reductase inhibitors, gene modification strategies, proteostasis regulator, or mRNA approaches will also need to consider the prenatal period, although ethical and practical concerns would accompany such intervention. It will be important also to consider at which stage in the postnatal period that any given form of intervention may have the greatest chance for success [58,71,72].

Other treatment options on the horizon include tissue or organ transplantation; for example, liver and in vivo or ex vivo gene therapy. The prospect of utilizing ovarian stem-cell based therapeutics for ovarian regeneration is also interesting. Such advances in reproductive medicine in the pipeline are bringing new hope to women diagnosed with POI from CG [73,74].

## 6. Conclusion

Despite several studies, the underlying pathophysiological mechanism and the timing of the ovarian dysfunction of POI from CG remain unclear. Substantial progress accomplished through exponential increase in knowledge in the field of infertility with better understanding of key signalling pathways and biomarkers offer exciting new discoveries and methods to preserve fertility in CG patients.

## Abbreviations

Akt	Protein kinase B
AMH	Anti-Mullerian hormone
CG	Classical Galactosaemia
FOX03	Forkhead box 03 gene
FSH	Follicle stimulating hormone
LH	Luteinizing hormone
Gal	Galactosaemia
Gal-1-P	Galactose-1-phosphate
GALT	Galactose-1-phosphate uridylytransferase
GALE	Galactose epimerase
GALK	Galactokinase
GALM	Galactose mutarotase
MTORC1	Mammalian target of rapamycin complex 1
P13K	Phosphatidylinositol 3-kinase
POI	Primary Ovarian Insufficiency
PTEN	Phosphatase and tensin homolog
UDP-Gal	Uridine diphosphate galactose
UGP	UDP-glucose pyrophosphorylase
